# In-vitro sonothrombolysis using thick-shelled polymer microbubbles - a comparison with thin-shelled microbubbles

**DOI:** 10.1186/s12947-020-00194-2

**Published:** 2020-05-04

**Authors:** Jovana Janjic, Malin K Larsson, Anna Bjällmark

**Affiliations:** 1 Biosense Webster, Johnson & Johnson Medical, Via del Mare 56, 00071 Pomezia, Rome, Italy; 2 Karolinska University Hospital, Eugeniavägen 3, SE-171 76 Stockholm, Sweden; 3 Department of Natural Science and Biomedicine, School of Health and Welfare, Jönköping University, Gjuterigatan 5, SE-553 18 Jönköping, Sweden

**Keywords:** Contrast agent, Cavitation, Human blood clot, Microbubble, Sonothrombolysis, Ultrasound

## Abstract

**Background:**

Vascular thrombosis can be treated pharmacologically, however, serious shortcomings such as bleeding may occur. Several studies suggest that sonothrombolysis can induce lysis of the clots using ultrasound. Moreover, intravenously injected thin-shelled microbubbles (MBs) combined with ultrasound can further improve clot lysis. Thick-shelled MBs have been used for drug delivery, targeting and multimodal imaging. However, their capability to enhance sonothrombolysis is unknown. In this study, using an in-vitro set-up, the enhancement of clot lysis using ultrasound and thick-shelled MBs was investigated. Thin-shelled MBs was used for comparison.

**Method:**

The main components in the in-vitro set-up was a vessel mimicking phantom, a pressure mearing system and programmable ultrasound machine. Blood clots were injected and entrapped on a pore mesh in the vessel phantom. Four different protocols for ultrasound transmission and MB exposure (7 blood clots/protocol) were considered together with a control test were no MBs and ultrasound were used. For each protocol, ultrasound exposure of 20 min was used. The upstream pressure of the partially occluded mesh was continuously measured to assess clot burden. At the end of each protocol blood clots were removed from the phantom and the clot mass loss was computed.

**Results:**

For the thick-shelled MBs no difference in clot mass loss compared with the control tests was found. A 10% increase in the clot mass loss compared with the control tests was found when using thin-shelled MBs and low pressure/long pulses ultrasound exposure. Similarly, in terms of upstream pressure over exposure time, no differences were found when using the thick-shelled MBs, whereas thin-shelled MBs showed a 15% decrease achieved within the first 4 min of ultrasound exposure.

**Conclusion:**

No increase in clot lysis was achieved using thick-shelled MBs as demonstrated by no significant change in clot mass or upstream pressure. Although thick-shelled MBs are promising for targeting and drug delivery, they do not enhance clot lysis when considering the ultrasound sequences used in this study. On the other hand, ultrasound in combination with thin-shelled MBs can facilitate thrombolysis when applying long ultrasound pulses with low pressure.

## Background

Ischemic heart disease and stroke, induced by vascular thrombosis, are the major causes of death in the high-income countries [1]. Administration of recombinant tissue plasminogen activator (rt-PA) is a common treatment method, however, recanalization rates using rt-PA are low, especially in patients with major proximal occlusions [2, 3] and approximately half of the rt-PA treated patients have unfavorable outcome in the long-term [4]. Additionally, significant side effects such as hemorrhage may occur during or after pharmacological thrombolysis. Hence, there is a need for easy applicable therapeutic strategies with a high recanalization rate and less serious side effects.

Sonothromolysis is a promising approach, and the capability of ultrasound to accelerate recanalization of thrombolytic occluded arteries during rt-PA treatment has been demonstrated [5]. It has also been shown that ultrasound alone facilitates thrombolytic therapy [6, 7]. The administration of ultrasound contrast agents, consisting of encapsulated microbubbles (MBs), has shown to further accelerate ultrasound-enhanced thrombolysis [8]. A number of potential mechanisms behind accelerated thrombolysis under the influence of ultrasound and MBs have been suggested such as acoustic cavitation, microstreaming, mechanical effects and local heating [6]. However, when considering acoustic cavitation - the probable main mechanism -, inconsistent results have been found [9–11]. The question is whether to use stable cavitation (i.e. MB oscillation) or induction of inertial cavitation or MB rupture, using higher acoustic pressures, for efficient thrombolysis. Furthermore, the effect of pulse length and excitation (center) frequency of the ultrasound is not fully known. However, it was shown that increased pulse length improved the lysis of blood clots and microemboli [12, 13].

Different MBs respond differently to ultrasound exposure, where for example thick-shelled MBs demonstrate a different acoustical and mechanical behavior compared with thin-shelled MBs due to differences in compressibility and visco-elastic properties. Typically, thick-shelled MBs oscillate and rupture at higher acoustic pressures than thin-shelled bubbles, and the rupture process is different for the two MBs [14]. Moreover, it has been shown that the efficiency of sonothrombolysis is greater when the applied ultrasound matches the natural resonance frequency of the MBs [15]. Thick-shelled MBs have higher resonance frequency, which in turn is dependent on factors such as size and shell properties. Most studies have used commercially available contrast agents with no ability to change MB properties. Therefore, only a limited number of studies have evaluated the influence of MB properties on thrombolytic efficiency [16]. Inconsistent results have been found of the influence of the gas contained within the MB [17, 18], whereas MB size and shell elasticity had a significant impact on thrombolytic efficacy [15].

The present generation of thin-shelled MBs have physical properties of the shell which limit their use. The new advancement with contrast agents containing thick-shelled MBs has a promising future where MBs can be used both as a substance carrier (i.e. inside the MB) and to incorporate substances into the shell; an advantage which is not possible in the present lipid-based ultrasound contrast agents. So far, it has been shown that thick-shelled polymer MBs can be used for multimodal imaging [19], for targeting [20] and for drug delivery [21]. However, there is a need to understand the potential of thick-shelled polymer MBs for sonothrombolysis. Therefore, the aim of this in-vitro study was to assess the capacity of thick-shelled polymer MBs to enhance the thrombolysis using different ultrasound sequences. The commercially available thin-shelled MBs was used for comparison. The thrombolytic enhancement was evaluated by measuring the clot weight reduction and the change in upstream pressure in a vessel-mimicking phantom.

## Methods

### In-vitro set-up and phantom construction

**Fig. 1:**
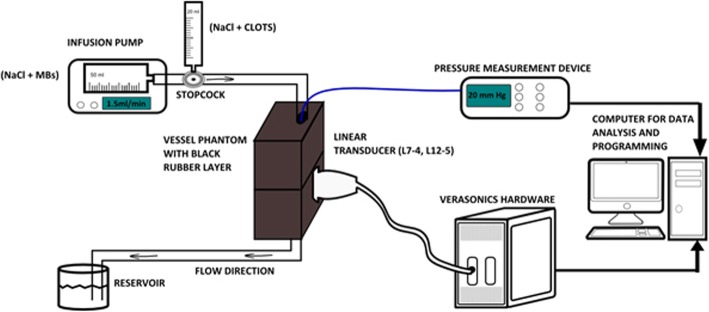
In-vitro set-up

The experimental set-up consisted of a vessel-mimicking phantom, a syringe pump, a pressure measuring device and a programmable ultrasound system (the Verasonics) connected to a computer (Fig. 1). The ultrasound transducer was fixed by a metallic holder and kept in contact with the phantom surface, which was covered with ultrasound gel. The transducer was positioned longitudinally to the vessel.

**Fig. 2:**
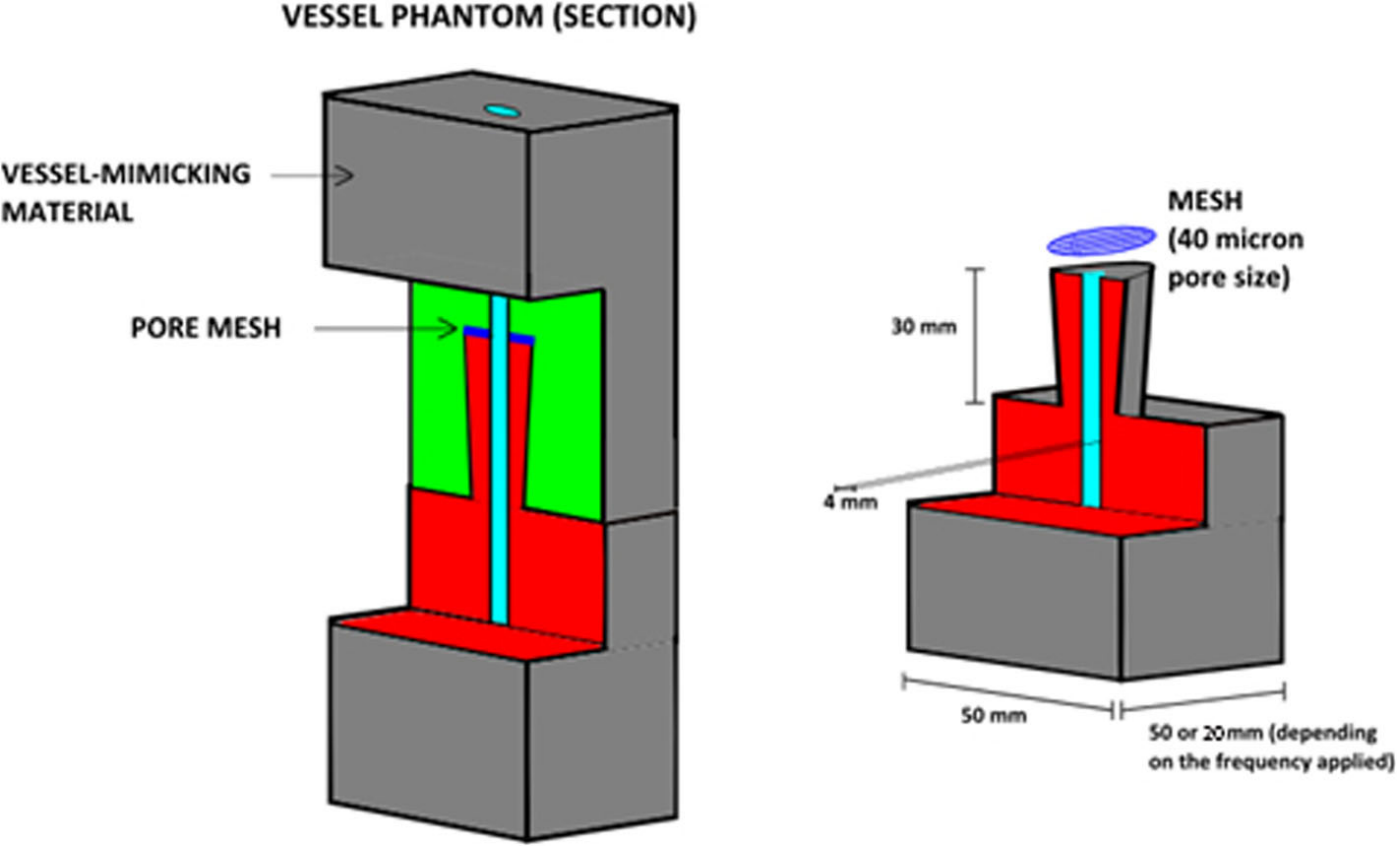
Vessel phantom

The vessel-mimicking material of the phantom was obtained by mixing 400 ml of deionized water with 15% of poly (vinyl alcohol) (PVA) and 3% of graphite. The PVA ensured mechanical characteristic similar to soft tissue and the graphite was added in order to reproduce scattering properties of tissues and tissue-like speckle texture [22]. The mixture of PVA, graphite and deionized water was stirred and heated up to 90 °C and then poured in two moulds made of delrin polyacetal and polymethyl methacrylate, designed in order to obtain the shape of a rectangular prism (Fig. 2) with a vessel lumen of 4 mm in size. When the moulds were completely filled with the mixture they were stored for 12 h at − 20 °C. After that, the phantom was kept at room temperature for another 12 h. This freeze-thaw cycle was repeated three times. Phantom characteristics was assessed by estimation of attenuation coefficient and speed of sound. For the estimation of attenuation coefficient, a flat sample (thickness 10 mm) of the phantom material was positioned in deionized water on a metal reflector. A single element transducer, delivering ultrasound at frequency of 2.25 MHz, was positioned opposite to the reflector and perpendicularly to the sample. The ultrasound was delivered using an ultrasonic pulser-receiver (Olympus, USA). The frequency spectrum was recorded using an oscilloscope (Tektronix, USA). The frequency spectrum was computed firstly with only the metal reflector without the sample and, secondly, with the metal reflector and the sample. The two spectra were than used for computing the attenuation coefficient knowing the distance of the transducer from the sample (10 cm) and the ultrasound velocity in water (1500 m/s). Assuming a linear dependence between the attenuation coefficient and the frequency, the final attenuation coefficient obtained was 0.5 dBcm^− 1^ MHz^− 1^. The speed of sound of the phantom was assessed using the same equipment as for the attenuation coefficient estimation. The speed of sound was estimated to 1510 m/s and was calculated by dividing the thickness of the phantom with the time difference between the echos from the front side and back side of the sample.

A 15 μm thick filter of nylon with 40 μm pores was prepared by removing the polypropylene frame from a cell strainer (Falcon Franklin Lakes, USA). The vessel phantom was composed of two halves, which were connected after positioning the 40 μm pore mesh between them (Fig. 2). This design kept the clot in position during the experiment, and enabled to change the mesh and to extract the remained clot after finishing the experiment. The thickness of the phantom varied depending on the ultrasound frequencies applied. When transmitting pulses at low frequency the transducer was placed 25 mm from the vessel lumen while the highest frequency (11.25 MHz) led to the need of a smaller distance, which was equal to 10 mm approximately. In this way, the ultrasound voltage in the clot area was reduced to the same value (43% of the transmitted voltage) for both the thicknesses assuming the attenuation coefficient equal to 0.5 dBcm^− 1^ MHz^− 1^.

An infusion syringe pump (Alaris, CareFusion, USA) with a flow velocity of 2 ml/min was used. The syringe (50 ml capacity) was connected to the plastic tube that delivered the flow to the phantom. The connector between the syringe and the pump had a stopcock (BD Medical, Unites States) with an aperture allowing the possibility to connect a second syringe. The second syringe (20 ml) was used for the injection of the clots. This injection procedure prevented the formation of air bubbles, which influenced the pressure measurements, within the system. After passing through the vessel, the flow solution was collected in a second reservoir. In order to keep the two halves of the phantom together, a system with a metallic rod was used with two movable and flat elements mounted on it. The two elements were regulated in order to keep the phantom in a stable vertical position.

### Pressure measurements

The set-up also included an ultra-miniature optical pressure transducer connected to the Samba 201/202 control unit (Life Science, Sweden) in order to continuously measure the upstream pressure. The pressure sensor was kept 4.5 cm above the mesh for all the experiments and the data were collected using the Samba software installed on a portable computer. The sample frequency of the Samba unit was 10 Hz and the measured pressure was the relative pressure, which was computed after calibration of the sensor against ambient pressure. This calibration procedure was performed before each test. The pressure curves were filtered using an average filter. Note that, due to leakages problem in the experimental setup, not all the pressure measurements were considered in the final analysis. If leakages between the two halves of the phantom were observed, the corresponding pressure measurement was discarded. In the final analysis, only three pressure measurements per protocol were considered.

### Clot preparation

The blood clots were produced using venous blood drawn from a healthy volunteer and stored in 4.5 ml tubes (BD Vacutainer, USA) containing 0.5 ml of citrate solution (0.105 M), which prevented the blood from coagulating. In order to produce clots, 750 μl of blood were mixed with 60 μl of CaCl_2_ solution (0,756 M) in plastic Eppendorf tubes [23]. The CaCl_2_ solution was prepared mixing deionized water with granular anhydrous CaCl_2_ (Sigma-Aldrich, USA) using a magnetic stirrer. The tubes containing the mixture of blood and CaCl_2_ were than incubated at room temperature for 3 h. The obtained clots were then cut in order to obtain a size comparable to the lumen diameter of the vessel (3-4 mm approximately). Before each test, the obtained clots were bottled on an absorbent paper and weighed on a 0.001 g precision scale (Sartorius, Germany). After each test the clots were extracted from the vessel (taking apart the two halves of the phantom), bottled and weighed again. The clot mass loss (%) was expressed as the difference between the initial and final weight divided by the mass before the test.

### Contrast agents

Two types of MBs were used in the study; the commercially available thin-shelled SonoVue MB (Bracco Imaging, Italy) and an in-house made thick-shelled MB. Their respective characteristics are displayed in Table 1. The production of the thick-shelled MBs was described in [24]. In short, 200 ml of MilliQ water were mixed with 4 g PVA (2%) and heated up to 80 °C. When the mixture reached the desired temperature, 380 mg of NalO4 were added and the solution was kept at 80 °C for 1 h continuously agitated with a magnetic stirrer. After 1 h the mixture underwent high shear stirring (8000 rpm for 3 h) in order to selectively split the head-to-head sequence contained in the PVA chains. This stirring was achieved using an Ultra Turrax (IKA, Germany) at room temperature. The resulting batch was washed seven times every 24 h. Concentration of the thick-shelled MBs was assessed using a light microscope and a counting chamber as described in the protocol presented by Pretzl and Cerroni [27]. The preparation of SonoVue MBs followed standard procedures where a saline solution (5 ml) and lyophilized powder (25 mg) were mixed for 20s. The two types of MBs were diluted in saline (NaCl) in order to have a concentration of 2x10^6^MBs/ml during the experiments.

**Table 1:** Overview of the characteristics of the contrast agents used in the study [24–26]

Contrast agent	Shell material	Gas	Diameter and shell thickness	Resonance frequency
Thick-shelled MBs (in-house made)	Polymer	Air	3.8 ± 0.6 μm 700 nm	12 MHz
Thin-shelled MBs (SonoVue)	Phospholipid	Sulfur hexaflouride	2.5 (1–10 μm) 4 nm	1–4 MHz

### Ultrasound transmission

**Fig. 3:**
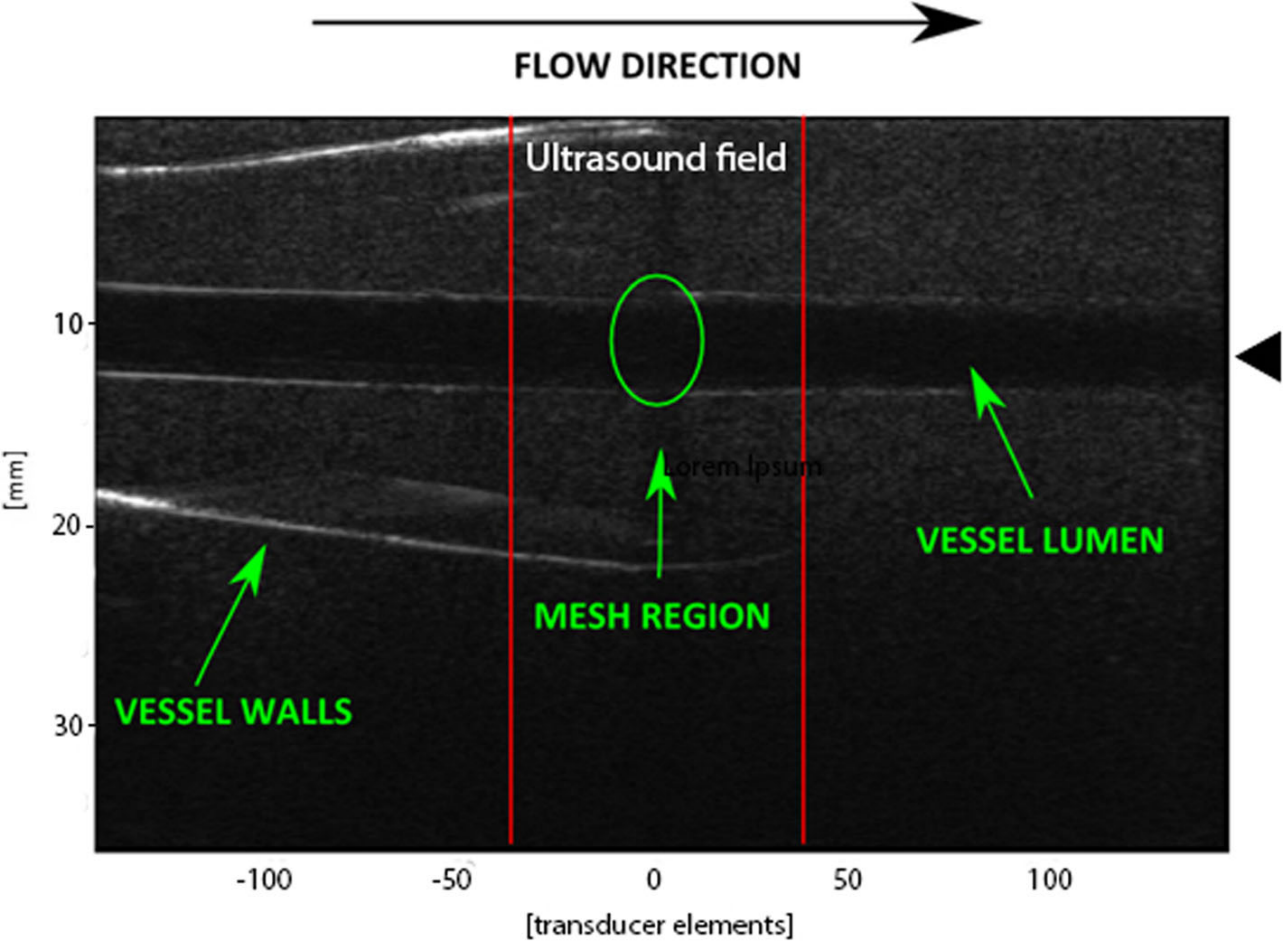
The ultrasound field using the L12–5 50 mm transducer

The ultrasound sequences were implemented using a Verasonics system (Verasonics, USA), which is a programmable ultrasound system. Two different transducers were used: L12–5 50 mm and L7–4 transducers (ATL/Philips, USA). In order to apply ultrasound with a frequency close to the resonance frequency of the two types of MBs, the L12–5 50 mm transducer transmitted at a frequency equal to 11.25 MHz (highest value achievable) and the L7–4 transducer transmitted at 4.09 MHz (lowest value achievable) were used. The focus of the ultrasound beam was set within the clot region, and ultrasound beam covered an area of 10–11 mm that included the clot and a small surrounding area, see Fig. 3.

The four protocols for ultrasound transmission are summarized in Table 2. For each of the protocols seven tests were performed. Moreover, seven control tests with no ultrasound and no MBs were used as reference.

**Table 2:** Characteristics of the ultrasound transmission protocols. MB = microbubble

Protocol	MB type	Frequency (MHz)	Voltage (V)	Pulse duration (ms)	Distance transducer/clot (mm)
Control test	–	–	–	–	–
1	Thick-shelled	11.25	100	5	10
2	Thick-shelled	4.09	100	5	25
3	Thin-shelled	4.09	100	5	25
4	Thin-shelled	4.09	50	10	25

For the four protocols, ultrasound exposure was performed for 20 min, starting directly after B-mode imaging verified that MBs were delivered to the clot area. Between each transmitted 5/10 ms ultrasound pulse, an idle time of 6 s was set allowing for MBs replenishment to the clot area given a flow velocity equal to 2 mm/s. Moreover, due to limitations in the Verasonics hardware each transmitted 5/10 ms pulse consisted of trains of pulses with the following characteristics: Protocol 1: pulse duration 88 μs, idle time 36 μs, Protocol 2–4: pulse duration 240 μs, idle time 7 μs. Imaging was not performed during the exposure of the clots to ultrasound and MBs, but once during the 6 s idle time to verify effective MB replenishment.

### Statistical analysis and data presentation

Mann-Whitney U-test was performed to test if there was a significant difference in mean mass loss of the blood clot between the control tests and the four protocols. The pressure curves were normalized to the first value, and the mean normalized pressure was plotted together with standard deviations bars. Normalization of the plots was necessary in order to allow comparisons due to the fact that slightly different values in the initial clot mass led to different initial pressure values.

## Results

Table 3 summarizes the initial mean weight and the mean mass loss, with the corresponding *p*-value, for the control tests and the four protocols. As can be seen in the Table 3, only thin-shelled MBs being exposed to low pressure/long pulses ultrasound exposure (protocol 4) showed a significant difference, by a 10% increase in the clot mass loss, compared to the control tests. When comparing the protocols for thick-shelled MB, no frequency-dependent variations in the clot mass loss was found.

**Table 3:** Initial mean weight (n = 7) and mean mass loss (*n* = 7) for each protocol (control tests included). NS = not significant, n = number of clots tested

Protocol	Initial mean weight (g)	Mean mass loss (%)	p-value
Control test	0.064 (SD 0.007)	38.0 (SD 9.6)	–
1	0.054 (SD =0.01)	37.0 (SD 9.7)	NS
2	0.063 (SD =0.01)	37.2 (SD 5.4)	NS
3	0.051 (SD =0.01)	35.8 (SD 6.3)	NS
4	0.063 (SD =0.01)	49.0 (SD 7.9)	0.025

**Fig. 4:**
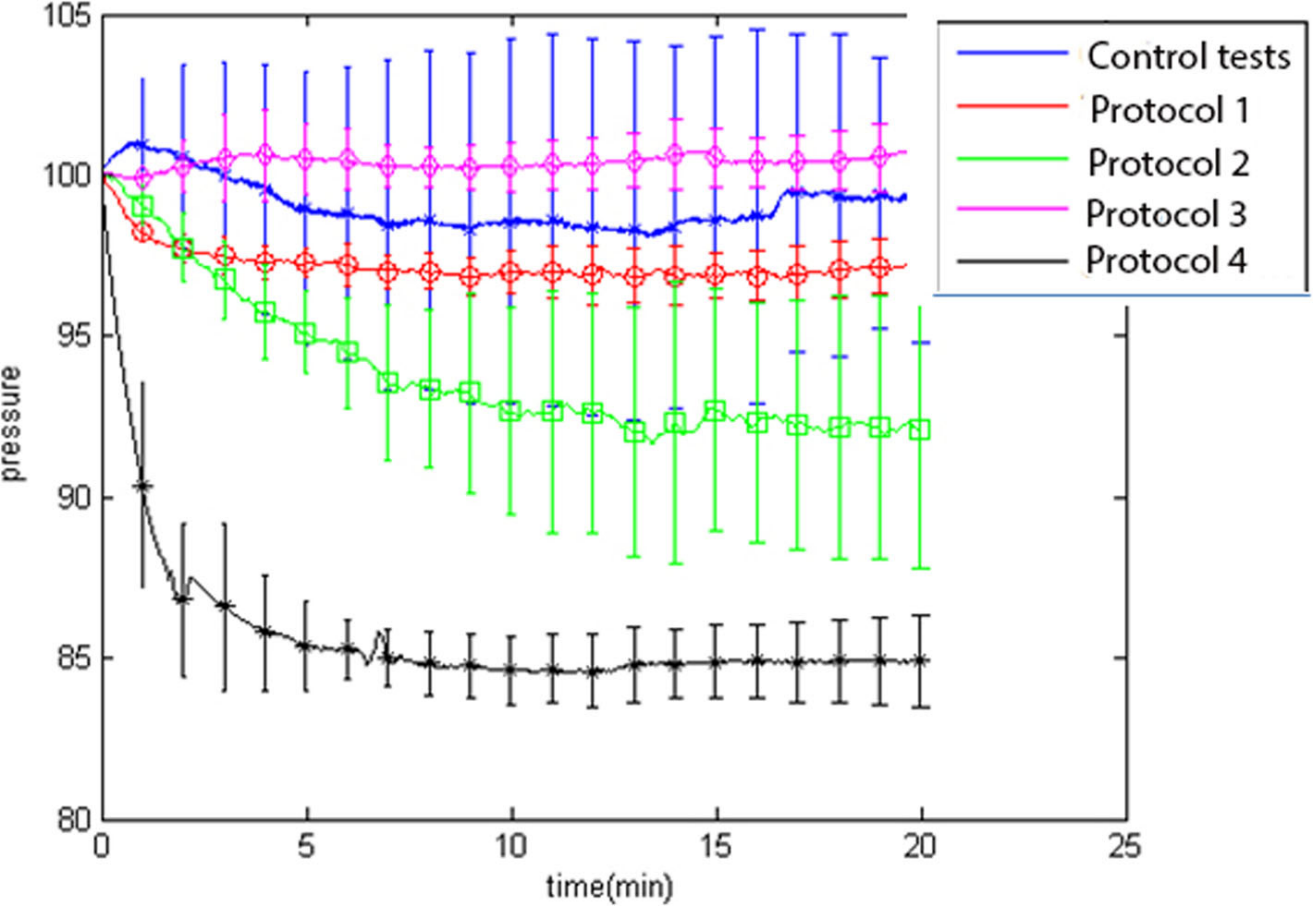
Mean normalized pressure plots for all protocols including control tests

Figure 4 presents the change in upstream pressure over exposure time. These results are in line with the result from the mean mass loss measurement by showing the highest impact on the thin-shelled MBs exposed to the low pressure/long pulses ultrasound (protocol 4).

## Discussion

The results of this study indicate that the tested thick-shelled MBs have limited ability to facilitate sonothrombolysis. It is well-known that thick-shelled MBs demonstrate a different acoustical behavior than thin-shelled MBs due to differences in compressibility and visco-elastic properties. The thick-shelled MBs are less flexible and higher pressure levels are needed to obtain non-linear oscillations and MB destruction [14]. One could assume that the fact that stable cavitation appears to be more effective for clot lysis compared to interial cavition [9, 15] should be favorable to the thick-shelled MBs, as they are more tolerable to high pressure levels. However, this was not seen in the present study. One possible explanation could be that the thicker and stiffer shell results in a considerable damping and a less well-defined resonance frequency making the oscillations of the MBs insufficient to facilitate clot lysis. The advantage of the thick-shelled MBs with their high mechanical and chemical stability [24], facilitating the use of the MBs as a carrier for different substances that can be incorporated into the shell or attached to the shell surface, implying that the future application for thick-shelled MBs is probably more related to targeting or a pharmaceutical delivery concept. Previous research has shown that MBs, tagged with antibodies or other ligands, can bind to components of a thrombus thereby increasing the concentration of MBs near thrombi in order to maximize sonothrombolysis [28, 29]. Moreover, encapsulated rt-PA combined with MBs can further improve thrombolytic efficacy. This can possibly reduce the injected dose of rt-PA thereby minimizing systemic exposure to rt-PA. Engineering of the MB surface and content with representative targeting ligands resulting in enhanced bioadhesion with cells for combined targeting and drug delivery is an appealing approach for the future, however, it should be kept in mind that modifications of MBs change their acoustic and mechanical behavior which further emphasizes the combined optimization of ultrasound output parameters and MBs properties. Enhanced biodegradability of the MBs might also be important for efficient in vivo drug release, especially for polymer-shelled MBs-.

The results also showed the thin-shelled MBs are sensitive to the chosen transmitting parameters of the ultrasound, as evidence by the difference in clot mass loss and pressure changes between the two protocols including thin-shelled MBs. The lower pressure levels in protocol 4 made it possible to program a train of pulses with longer duration (10 ms), and both lower pressure levels facilitating stable cavitation and longer pulses have shown to be beneficial in clot lysis [9, 13]. Moreover, mean pressure decrease (Fig. 4) was characterized by an early decrease in the first 4 min, followed by a plateau region. The early decrease is in accordance with previous results, where the maximal thrombolytic effect was seen within the first 3 min [12]. Possibly the clot lysis could have be more pronounced if the resonance frequency of SonoVue was perfectly targeted.

Clot mass loss was seen for all the protocols (control tests included). However, this was not the case for the pressure measurement where decreased values were mainly seen for the thin-shelled MBs exposed to low pressures. One possible explanation could be that the saline flow produced clots debris which remained entrapped within the mesh, hence not allowing the flow to be restored and keeping the pressure to high values. A similar problem is also seen in the clinics where methods focusing on opening occluded arteries do not achieve optimal microvascular perfusion [30]. Ultrasound in combination with MBs has shown potential to enhance microvasular perfusion where the opening microvascular collaterals or the lysis of microemboli have been suggested as possible explanations of this effect [12, 31]. For future application, it is of great interest to identify if different settings in terms of ultrasound and MB parameters are optimal for the lysis of clots and microemboli, respectively. Moreover, for in-vitro application similar to the present study, the efficiency of the clot lysis should be computed considering also the clot debris stuck in pore mesh. By developing a method for analyzing the material entrapped in the mesh, it would be possible to fully understand if the obtained pressure values with its relatively high variability could be explained by the fact that that clot debris occasionally was bigger in size than the mesh pores.

To obtain efficient sonothrombolysis, also optimization of MB concentration needs to be considered in future applications. A previous study showed maximal sonothrombolysis at MB concentrations > 10^8^ MB/ml and that the concentration is dependent on MB diameter [15]. The concentration of MBs used in this study (2 × 10^6^ MB/ml) was lower than the above-mentioned study, and potentially a higher concentration could have yielded a more pronounced effect on the obtained results. On the other hand, this concentration was higher than the recommendations for clinical contrast administration [32]. Even though contrast administration is associated with low incidence of adverse events [32], the patient safety perspective must be taken into account when sonothrombolysis is introduced into clinical practice.

The experimental set-up had some limitations. There were problems with leakages which affected the number and quality of pressure measurements, making them less reliable as compared with the clot mass computations. Also, the disregarding of pressure measurements based on visual assessment can potentially introduce bias in the results. Furthermore, the Verasonics system had some limitations in the coding and implementation of different combinations of ultrasound parameters due to the fact that the entire system had power limitations and hardware constraints that did not allow realization of continuous long pulses. In the present study, the 5/10 ms ultrasound pulse was composed by several shorter pulses with small idle times in between. Using the L12–5 transducer, the longest pulse achievable with 11.25 MHz had a duration of 88.18 μs, while the L7–4 transducer transmitting at 4.09 MHz enabled pulses with maximal duration of 240 μs. Thus, in order to obtain a longer pulse, several of these short pulses were combined. The idle time in between was the lowest achievable and smaller values were not possible due to power limitations. Moreover, each pulse had a duty cycle equal to 37%. The different characteristics between the two transducers used in the study limit direct comparison between protocol 1 and protocol 2–4.

The experimental set-up in-vitro presented several aspects similar to the physiological situation of occluded coronary arteries. The vessel lumen was 4 mm in size, comparable to the dimension of the left anterior descending coronary artery. Moreover, the 40 μm pore mesh reproduced the microcirculation and the flow velocity was 2 mm/s, similar to the real situation. On the contrary, the distance between the clot region and the ultrasound transducer was not comparable to the real distance between the probe and the coronary arteries. Moreover, in a clinical situation the presence of the ribs and other body structures can attenuate and scatter the ultrasound waves which limits any usage of linear transducers. Another limitation of the in-vitro set-up was the continuous flow delivery, which is not present in the real situation where the flow is pulsatile. However, the continuous flow limited to formation of air bubbles in the set-up, thereby eliminating random peaks in the pressure patterns.

## Conclusion

Thick-shelled MBs, with great potential for targeting and drug delivery, demonstrate no increase in clot lysis for the tested ultrasound sequences. Ultrasound in combination with thin-shelled MBs can facilitate thrombolysis as evidenced by an increase in the clot mass loss and a decrease in upstream pressure when applying long ultrasound pulses with low pressure.

## Data Availability

The datasets used and/or analysed during the current study are available from the corresponding author on reasonable request.

## Abbreviations

MB: Microbubble
PVA: Poly (vinyl alcohol)
rt-PA: Recombinant tissue plasminogen activator
